# Supplementary material for: Harmonic oscillator based particle swarm optimization
Source: PLoS One. 2025 Jun 27;20(6):e0326173. doi: 10.1371/journal.pone.0326173 (PMC12204584; doi:10.1371/journal.pone.0326173)
Supplement: S1 — (PDF) [file pone.0326173.s001.pdf]

S1 Black-box benchmarking

One might argue that the superior performance of HOPSO on the selected test functions could be due to a bias toward functions with minima at zero or similar characteristics. To address this concern and validate the generality of our approach, we further evaluated the HOPSO algorithm on a well-established black-box benchmarking suite, COmparing Continuous Optimizers (COCO) [1].

COCO is a comprehensive benchmarking framework designed to evaluate the performance of optimization algorithms on a diverse set of continuous optimization problems. The suite includes a wide range of test functions, spanning both unimodal and multimodal problems, as well as noisy and ill-conditioned landscapes, making it an ideal benchmark for assessing the robustness and versatility of optimization methods. For more details on test functions and their modified version, please refer to [1]. By applying HOPSO to COCO, we aimed to test its ability to perform across various problem domains beyond those initially chosen for our experiments.

COCO post processing analysis was performed for all test functions using 15 instances with the maximum evaluations budget of 10000 $\times$ dimension. A summary of results for 51 different instances is shown in the Figs. 1–5 for 2,3,5,10 and 20 dimensions respectively. In COCO we evaluate each test function over 15 randomized instances–each a different shift and/or rotation of the base function–and we measure performance by the number of evaluations needed to reach a predefined sequence of target accuracies (called function–target pairs). These performance-profile plots display, for each optimizer, the fraction of benchmark targets reached as a function of computational effort (log_{10} of function-evaluations per dimension). Each solid curve rises when the method first solves an additional target within the allotted budget (10000 $\times D$ evaluations), so a steeper initial climb indicates faster convergence on the easier targets. To interpret the plot, note (1) where each curve first ascends (how many evaluations/dim are needed to solve a given percentage of targets) and (2) the height reached at $log_{10}(evals/dim) = 4$ (the final success rate within budget).

Across all dimensions, HOPSO consistently achieves the highest success fractions within the fixed budget, closely followed by standard PSO. Both HOPSO and PSO rapidly ascend their curves, solving over 80% of targets by $\log_{10}(\text{evals/dim}) \approx 3.5$ in low dimensions, and retaining the best performance as dimension increases. DE exhibits moderate performance, with its curve climbing more slowly and plateauing around 40-50% success by the budget line. COBYLA lags behind all population-based methods, rarely exceeding 30% success even in 2-D and dropping below 10% in high dimensions. As dimensionality grows from 2 to 20, all algorithms require more evaluations per dimension (curves shift right) and solve fewer targets (lower plateau), but HOPSO's superior exploration and damping strategy yields the most robust scaling under tight evaluation limits.

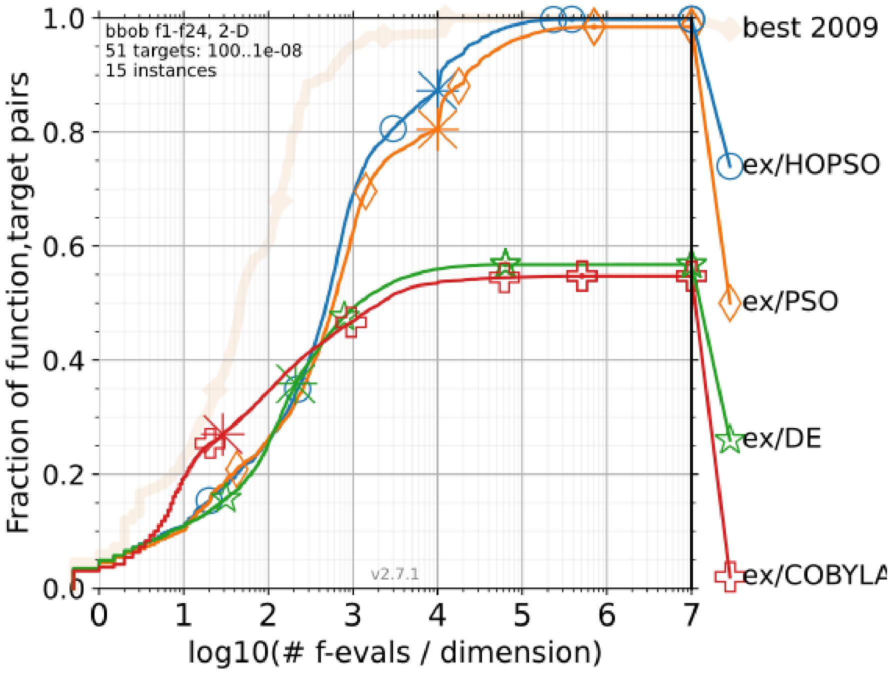

Figure 1: COCO performance on 2-D functions (budget = 20000 evals). HOPSO (blue) and PSO (orange) solve over 90% of targets by $\log_{10}(\text{evals/dim}) \approx 4$, DE (green) reaches $\approx$ 55%, and COBYLA (red) remains below 40%.

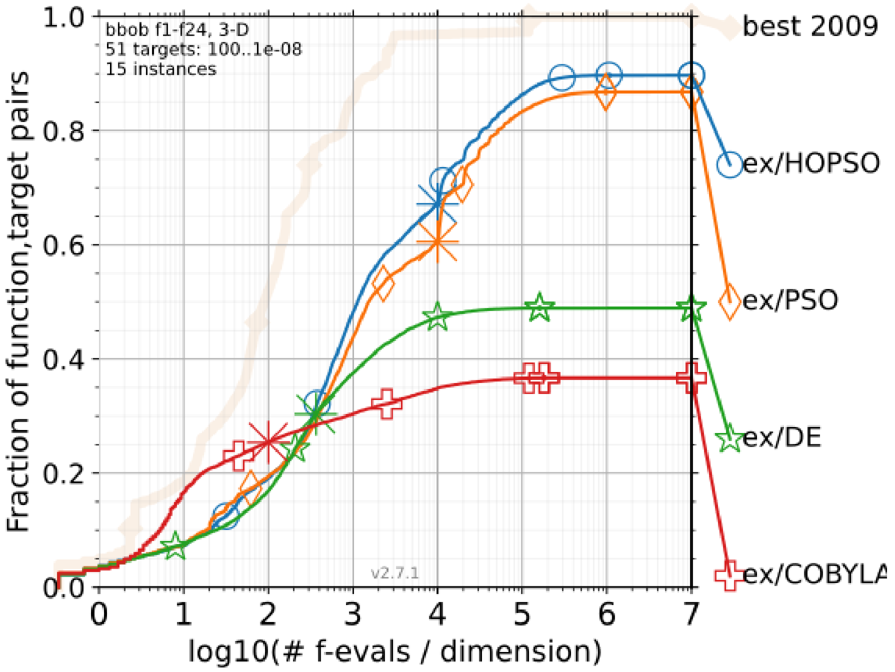

Figure 2: COCO performance on 3-D functions (budget = 30000 evals). HOPSO and PSO achieve >85% success by the budget line, DE plateaus near 45%, and COBYLA near 35%. HOPSO slightly outperforms PSO on the hardest targets.

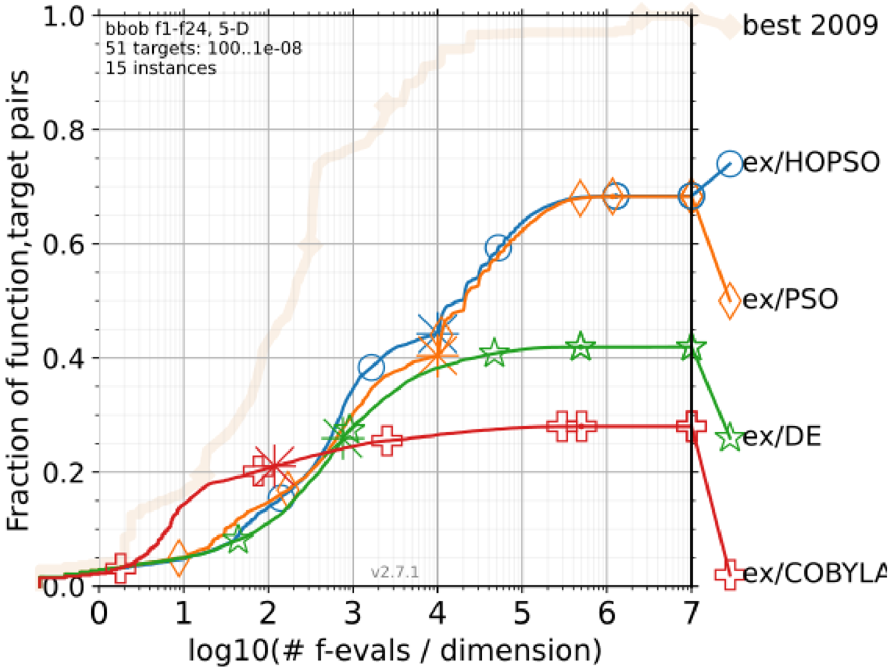

Figure 3: COCO performance on 5-D functions (budget = 50000 evals). HOPSO maintains $\approx$ 70% success, PSO $\approx$ 65%, DE $\approx$ 40%, and COBYLA $\approx$ 25%. The performance gap widens with dimensionality.

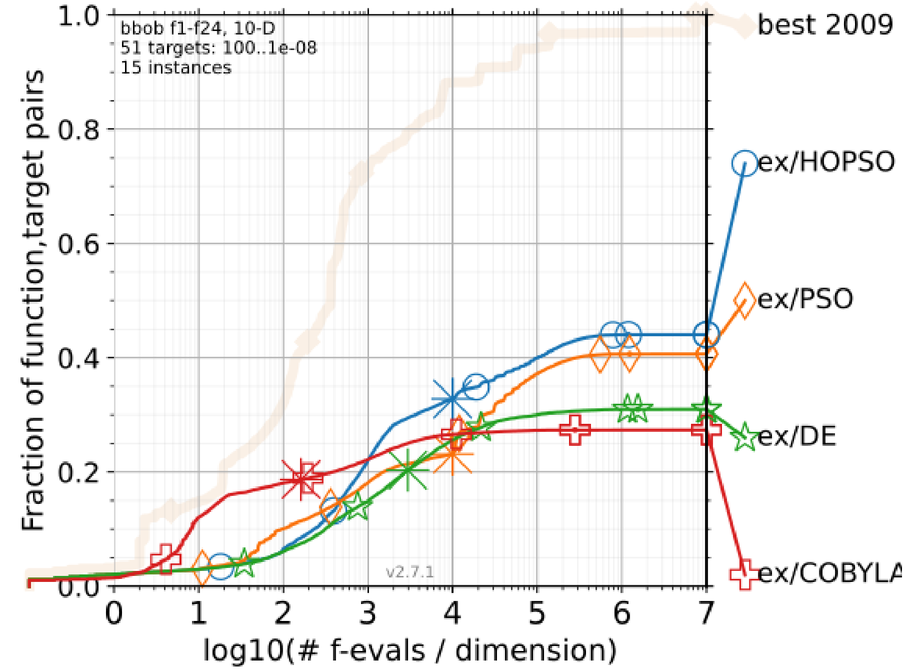

Figure 4: COCO performance on 10-D functions (budget = 100000 evals). HOPSO ($\approx$ 45% solved) and PSO ($\approx$ 40%) outperform DE and COBYLA (both $\approx$ 25%). All methods slow as dimension grows, but HOPSO remains most reliable.

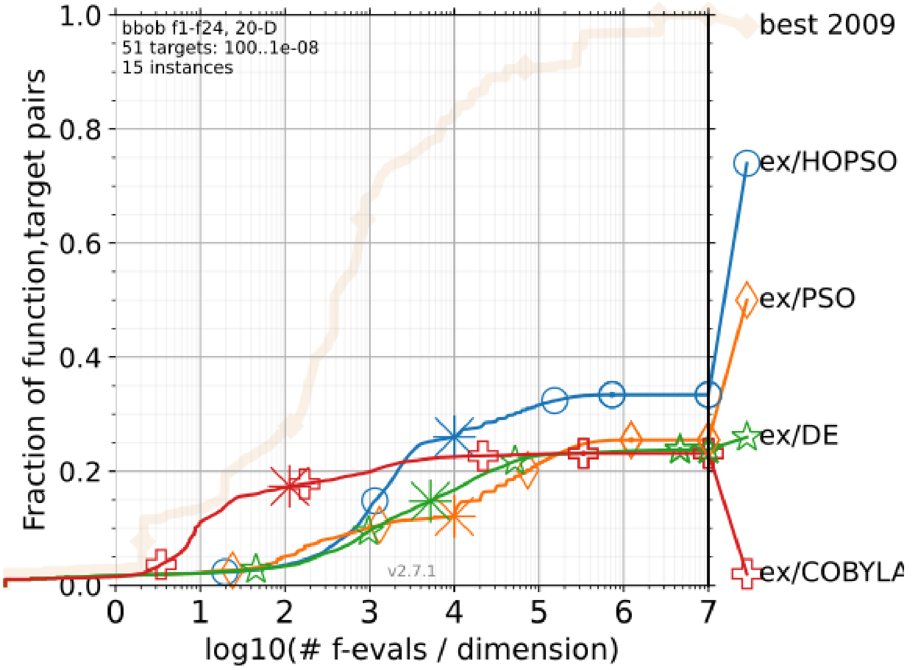

Figure 5: COCO performance on 20-D functions (budget = 200000 evals). HOPSO solves $\approx$ 30% of targets, PSO $\approx$ 25%, DE $\approx$ 20%, and COBYLA $\approx$ 5%. High dimensionality degrades all algorithms, yet HOPSO is the most robust under tight budgets.

References

[1] N. Hansen et al. “COCO: A Platform for Comparing Continuous Optimizers in a Black-Box Setting”. In: *Optimization Methods and Software* 36 (1 2021), pp. 114–144. DOI: `https://doi.org/10.1080/10556788.2020.1808977`.
