# Supplementary material for: Harmonic oscillator based particle swarm optimization
Source: PLoS One. 2025 Jun 27;20(6):e0326173. doi: 10.1371/journal.pone.0326173 (PMC12204584; doi:10.1371/journal.pone.0326173)
Supplement: Attachment — Submitted filename: Response to Reviewers_2.pdf [file pone.0326173.s002.pdf]

Dear Reviewers,

We appreciate your thoughtful and constructive comments on our manuscript, **"Harmonic Oscillator based Particle Swarm Optimization"** (PONE-D-25-05664). We have carefully considered suggestions and revised the manuscript accordingly. Below, we provide a detailed response to each suggestion. Changes made in the manuscript are written in red for clarity.

Reviewer #1

1) We have revised the paragraph to clarify that QD algorithms do aim to optimize a single objective function, but they also focus on finding diverse solutions across a feature space. We corrected the earlier confusion about where QD algorithms operate–they evolve solutions in parameter space while organizing them in feature space. We also fixed the typo (“feaure” → “feature”) and updated the references to cite only those that actually discuss QD methods. We appreciate the comments, which helped us improve the accuracy and clarity of this section.

2) As for the COCO analysis, we included all the results in the previous rebuttal letter.

   As suggested, we have decided to include the COCO-graphs in the supplementary material without changing the structure of the main article.

3) As for the code published on Github, we stress that the code has only a form of a supplementary material for this manuscript - this is now clearly mentioned in the readme file.

Faithfully

The Authors
